# Association between PaO_2_/FiO_2_ ratio and thrombotic events in COVID-19 patients

**DOI:** 10.1007/s11739-023-03196-w

**Published:** 2023-01-18

**Authors:** Lorenzo Loffredo, Pasquale Pignatelli, Matteo Pirro, Giancarlo Ceccarelli, Alessandra Oliva, Enrico Maggio, Francesca Cancelli, Damiano D’Ardes, Maria Amitrano, Anna Maria Zicari, Bianca Laura Cinicola, Gloria Taliani, Roberto Cangemi, Miriam Lichtner, Marco Falcone, Federica Orlando, Francesco Pugliese, Mario Venditti, Claudio Maria Mastroianni, Francesco Violi, COVID-Group, Fausto D’Agostino, Felice Eugenio Agrò, Alessia Mattei, Loredana Tibullo, Maria Grazia Nunziata, Valeria Iorio, Natalia Iuliano, Sara Mangiacapra, Mariangela Raimondo, Mariangela Atteno, Claudio Ferri, Davide Grassi, Giovambattista Desideri, Stefano Abballe, Serena Dell’Isola, Monica Rocco, Daniela Alampi, Cosmo Del Borgo, Vanessa Bianconi, Massimo Raffaele Mannarino, Filippo Figorilli, Alessia Fallarino, Ilaria Maria Palumbo, Arianna Pannunzio, Arianna Magna, Chiara Bagnato, Alba Rosa Alfano

**Affiliations:** 1 Department of Clinical Internal, Anesthesiologic and Cardiovascular Sciences, Sapienza University of Rome, Viale del Policlinico, 155, 00161 Rome, Italy; 2 Unit of Internal Medicine, Department of Medicine, University of Perugia, Perugia, Italy; 3 Department of Public Health and Infectious Diseases, Sapienza University of Rome, Piazzale Aldo Moro 5, 00185 Rome, Italy; 4 Clinica Medica, Department of Medicine and Aging, “G. D’Annunzio, University of Chieti-Pescara, Chieti, Italy; 5 Internal Medicine Unit, Moscati Hospital, Avellino, Italy; 6 Department of Maternal Sciences, Sapienza University of Rome, Viale Regina Elena, 324, 00161 Rome, Italy; 7 Department of Translational and Precision Medicine, Sapienza University of Rome, Rome, Italy; 8 Infectious Diseases Unit, Department of Clinical and Experimental Medicine, University of Pisa, Pisa, Italy; 9 Mediterranea Cardiocentro-Napoli, Via Orazio, 2, 80122 Naples, Italy

**Keywords:** Albumin, P/F ratio, COVID-19, Thrombosis

## Abstract

PaO_2_/FiO_2_ (P/F ratio) is considered a marker of hypoxia/hypoxemia and mortality. Several prothrombotic changes are associated with the decrease of P/F ratio. The role of P/F ratio in patients with arterial and venous thrombosis remains unclear. The aim of this study was to assess in patients with coronavirus disease 2019 (COVID-19), the association between P/F ratio and arterial/venous thrombosis. One thousand and four hundred and six COVID-19 patients were recruited; 289 (21%) patients had P/F ratio < 200 and 1117 (79%) ≥ 200. Compared to the patients with P/F ratio ≥ 200, those with P/F ratio < 200 were older and with higher levels of glycemia, D–dimer and lower levels of albumin. Multiple linear regression analysis showed that albumin (standardized coefficient *β*:  0.156; SE: 0.001; *p* = 0.0001) and D–dimer (standardized coefficient *β*: -0.135; SE: 0.0001; *p* = 0.0001) were associated with P/F ratio. During the hospitalization 159 patients were transferred in intensive care unit (ICU), 253 patients died, 156 patients had arterial or venous thrombotic events. A bivariate logistic analysis was performed to analyze the predictors of thrombosis in COVID-19 patients; P/F ratio < 200 (Odds Ratio: [OR] 1.718, 95% Confidence Interval [CI] 1.085–2.718, *p* = 0.021), albumin (OR 1.693, 95% CI 1.055–2.716, *p* = 0.029), D-dimer (OR 3.469, 95% CI 2.110–5.703, *p* < 0.0001), coronary artery disease (CAD) (OR 1.800, 95% CI 1.086–2.984, *p* = 0.023) and heart failure (OR 2.410 95% CI 1.385–4.193, *p* = 0.002) independently predicted thrombotic events in this population. This study suggests that the P/F ratio is associated with thrombotic events by promoting a hypercoagulation state in patients hospitalized for COVID-19.

## Introduction

COVID-19 is a pandemic associated with increased risk in the development of arterial and venous thrombotic events and death [1, 2]. The risk factors associated with the increased thrombotic risk are still unclear. Since COVID-19 is a disease that can cause pneumonia and therefore seriously affecting the respiratory function, some studies analyzed the role of the PaO_2_/FiO_2_ ratio (P/F ratio) and its impact on clinical outcome. P/F ratio is the ratio between arterial partial pressure of oxygen (PaO2) and fraction of inspired oxygen (FiO_2_) and is a marker of systemic hypoxia. It is currently used as a marker of acute respiratory failure in COVID-19 patients [3] and in adult patients with acute respiratory distress syndrome (ARDS) [4]. A P/F < 200 is considered a cutoff associated with a serious risk of developing ARDS and respiratory failure [5].

Several previous studies showed that a low P/F ratio is associated with a higher risk of mortality [3, 6] but there is scarce evidence of its association with thrombotic events in COVID-19 patients. However, it is arguable that the P/F ratio could be associated with thrombosis as animal and human studies have shown that hypoxia is strongly associated with thrombosis through mechanisms that activate platelets, increase levels of tissue factor, clotting factors or impair fibrinolysis [7].

For its simplicity, rapidity of execution and low cost the P/F ratio could represent a valid instrument to stratify the risk of developing thrombotic events in patients affected by COVID-19. Thus, in this study we want to assess if P/F ratio is associated with arterial and venous thrombotic events in COVID-19 patients.

## Materials and methods

### Study design and population

This is an observational retrospective cohort multi-center study performed in Italian Hospitals devoted to COVID-19 care. This study was performed in non-intensive care unit (ICU) medical wards. We enrolled consecutive patients, according to the inclusion/exclusion criteria form cohort from Rome Hospitals and from Aquila, Chieti, Latina, Perugia, Avellino and Viterbo.

We included in the study adult (≥ 18 years) patients with laboratory-confirmed COVID-19 and acute respiratory syndrome coronavirus-2 (SARS-CoV2)-related pneumonia which needed hospitalization, requiring or not mechanical ventilation, consecutively admitted to Medicine wards from March 2020 to March 2021. COVID-19 was diagnosed on the basis of the WHO interim guidance [8]. COVID-19 pneumonia was diagnosed by chest computed tomography. A COVID-19 case was defined as a person with laboratory confirmation of COVID-19 infection, irrespective of clinical signs and symptoms. Each hospital involved in this study recorded COVID-19 cases and forwarded the data to the national health ministry. During the intra-hospital stay any vascular event was registered. Demographic and clinical characteristics were collected after receiving informed consent. Routine analysis included P/F ratio, D-dimer and albumin executed within 48 h from the admission at the hospital. Ethical approval for this study was obtained from Ethics Committee of Azienda Ospedaliero Universitaria Policlinico Umberto I (ID Prot. 109/2020).

### Baseline assessment

Demographic and anamnestic characteristics, along with baseline clinical, laboratory and radiological results were extracted from electronic medical records of patients enrolled. Data regarding demographic characteristics, comorbidities, and concurrent therapy were collected. Pre-existence of diabetes mellitus, hypertension, cardiovascular disease, chronic kidney disease, and obesity were defined as previously described [9].

### Primary end-points

Arterial and venous thrombosis occurring during the hospitalization were the primary end-point of the study. The appearance of new ischemic/embolic events was diagnosed as follows: (1) pulmonary thromboembolism by lung CT scan; [10] (2) myocardial infarction by EKG changes associated with enhanced markers of cell necrosis; [11] (3) acute brain ischemia by onset of new focal neurological signs and symptoms and confirmed, whenever possible, by NMR or CT imaging; [12] (4) deep venous thrombosis in symptomatic patients was diagnosed by compression ultrasonography [13–15].

Fatal myocardial infarction was defined as previously described by the World Health Organization [16]. Microembolization of the clots originated in the heart or other arterial districts distant to the artery lower limbs responsible of arterial occlusion and ischemia where diagnosed as previously described [17].

### Statistical analysis

Data are expressed as mean and standard deviation (SD) or median and interquartile range (IQR) according to their distribution for continuous variables, and count and percentages for categorical ones.

Comparison between groups was performed by Chi square test, Student *T* test, or Mann–Whitney test as appropriate. Bivariate analysis was performed by Pearson's Correlation Coefficient. The variables with evidence of an association *p* < 0.10 were included in a multivariable linear regression using an automated procedure with forward selection. The results of the multivariable linear regression analysis were expressed as standardized coefficient beta (*β*) with standard error (SE), moreover the coefficient of determination was provided (*R*^*2*^). A *p* value of < 0.05 was considered statistically significant. A multivariable logistic regression model was estimated, and the odds-ratios obtained were used to derive weighting factors of thrombosis. We used the following cut-offs for D–dimer and albumin: D–dimer ≥ 1100 ng/mL and albumin < 35 g/L for chi-square and logistic regression analysis, while we treated them as continuous variables in bivariate correlation and multiple linear regression analysis.

All tests were two-tailed, and a value of *p* < 0.05 was considered as statistically significant. Analyses were performed using IBM SPSS statistic version 25.

## Results

One thousand and four hundred and six COVID-19 patients were recruited; 289 (21%) patients had P/F ratio < 200 and 1117 (79%) ≥ 200. The mean period of hospitalization was 19 ± 18 days. At admission, two hundred and one patients were already treated with anticoagulants.

Compared to the patients with P/F ratio ≥ 200, those with P/F ratio < 200 were older, with a major need of intensive care unit (ICU) and with a higher prevalence of smoking, coronary artery disease (CAD), thrombotic events and dementia (Table 1). Furthermore, they have higher levels of glycemia, D–dimer, IMPROVEDD score and lower albumin. Finally, patients with P/F < 200 ratio had a significant higher prevalence of steroids and heparins low-molecular-weight heparins (LMWH) use (Table 1).

**Table 1:** Clinical characteristics of COVID-19 patients with P/F ratio < 200 and P/F ratio ≥ 200

	P/F ratio < 200	P/F ratio ≥ 200	*p*
*N*.	289	1117	–
Age (years)	72 ± 13	65 ± 18	** < 0.0001**
Male	137/289 (47.4%)	536 /1117 (47.9%)	0.860
Obesity	25/99 (25.3%)	48/240 (20%)	0.285
Active smoking	28/122 (23%)	63/467 (13.5%)	**0.010**
Former smoking	4/33 (12.1%)	25/245 (10.2%)	0.735
Diabetes	51/215 (23.7%)	140/730 (19.2%)	0.145
Hypertension	138/240 (57.5%)	509/986 (51.6%)	0.227
Dyslipidemia	13/93 (14%)	53/233 (22.7%)	0.075
CAD	52/255 (20.4%)	141/1041 (13.5%)	**0.006**
Heart failure	33/157 (21%)	130/658 (19.8%)	0.722
Peripheral arterial disease	19/176 (10.8%)	84/745 (11.3%)	0.856
Previous venous thromboembolism	4/126 (3.2%)	11/440 (2.5%)	0.678
Atrial fibrillation	24/172 (14%)	110/902 (12.2%)	0.522
Previous TIA/stroke	15/244 (6.1%)	53/992 (5.3%)	0.621
COPD	30/227 (13.2%)	102/976 (10.4%)	0.230
Dementia	29/155 (18.7%)	79/637 (12.4%)	**0.040**
Cancer in the last 5 years	17/195 (8.7%)	62/710 (8.7%)	0.964
Thrombotic events	55/289 (19%)	101/1110 (9.1%)	** < 0.0001**
Intensive care unit	97/273 (35.5%)	62/999 (6.2%)	** < 0.0001**
LMWH	237/289 (82%)	706/1117(63.2%)	** < 0.0001**
Steroids	144/289 (49.8%)	350/1117 (31.3%)	** < 0.0001**
CRP (mg/L) (median [IQR])	18 [9–42]	17 [9–40]	0.073
D-dimer ≥ 1100 (ng/mL)	168/264 (63.6%)	346/936 (40%)	** < 0.0001**
Albumin < 35 (g/L)	192/265 (72.5%)	362/973 (37.2%)	** < 0.0001**
Glycemia (mg/dL)	137 ± 60	121 ± 49	** < 0.0001**
Creatinine (mg/dL)	1.36 ± 1.49	1.17 ± 1.38	0.063
IMPROVEDD	2.6 ± 1.3	2.1 ± 1.2	** < 0.0001**

Bold values indicates statistically significant

Bivariate correlation analysis showed that P/F ratio correlated with serum albumin (*R*: 0.227, *p* < 0.0001), D-Dimer (*R*: − 0.240 *p* < 0.0001), age (*R*: − 0.347, *p* < 0.0001), SpO_2_ (*R*: 0.258, *p* < 0.0001) in the overall population; no correlation was observed between P/F ratio and serum creatinine (*R*: − 0.022, *p* = 0.476). A significant inverse correlation was detected between D-dimer and serum albumin (*R*: − 0.206, *p* < 0.0001).

Multiple linear regression analysis showed that albumin (standardized coefficient *β*:  0.156; SE: 0.001; *p* = 0.0001), D-dimer (standardized coefficient *β*: -0.135; SE: 0.0001; *p* = 0.0001), were the independent predictive variables associated with P/F ratio (*R*^*2*^ = 30%).

During the hospitalization 159 patients were transferred in ICU, 253 patients died, 156 patients had the following thrombotic events: micro-embolism *n* = 5 (3%), deep venous thrombosis *n* = 42 (27%), pulmonary embolism *n* = 36 (23%), peripheral artery ischemia *n* = 9 (6%), myocardial infarction *n* = 36 (23%), transient ischemic attack (TIA)/stroke *n* = 28 (18%) (Table 2).

**Table 2:** Clinical characteristics of COVID-19 patients with and without cardiovascular thrombotic events

	Thrombotic event	No thrombotic event	*p*
*N*.	156	1243	–
Age (years)	76 ± 12	66 ± 17	** < 0.0001**
Male	82/156 (52.5%)	588/1243 (47.3%)	0.215
Obesity	8/56 (14.3%)	65/283 (23%)	0.149
Active smoking	18/86 (20.9%)	73/503 (14.5%)	0.128
Former smoking	7/26 (26.9%)	22/252 (8.7%)	**0.004**
Diabetes	28/132 (21.2%)	163/813 (20.1%)	0.758
Hypertension	97/141 (68.8%)	545/1078 (50.6%)	** < 0.0001**
Dyslipidemia	13/56 (23.2%)	53/270 (19.6%)	0.543
CAD	38/138 (27.5%)	153/1151 (13.3%)	** < 0.0001**
Heart failure	39/113 (34.5%)	124/702 (17.7%)	** < 0.0001**
Peripheral arterial disease	19/89 (21.4%)	83/825 (10.1%)	**0.004**
Previous venous thromboembolism	6/77 (7.8%)	9/489 (1.8%)	**0.003**
Atrial fibrillation	22/115 (19.1%)	110/952 (11.6%)	**0.020**
Previous TIA/stroke	12/131 (9.2%)	56/1088 (5.1%)	0.055
COPD	27/134 (20.2%)	101/1062 (9.5%)	** < 0.0001**
Dementia	13/73 (17.8%)	91/712 (12.8%)	0.228
Cancer in the last 5 years	9/84 (10.7%)	68/814 (8.4%)	0.516
P/F ratio < 200	55/156 (35.3%)	234/1243 (18.8%)	** < 0.0001**
Intensive care unit	35/135 (25.9%)	124/1131 (11%)	** < 0.0001**
LMWH	112/156 (71.8%)	830/1243 (66.8%)	** < 0.0001**
Steroids	47/156 (30.1%)	446/1243 (35.9%)	** < 0.0001**
CRP (mg/L) (median [IQR])	18[12–41]	17 [8–40]	0.070
D–dimer ≥ 1100 (ng/mL)	94/135 (69.6%)	418/1060 (39.4%)	** < 0.0001**
Albumin < 35 (g/L)	99/144 (68.8%)	452/1090 (41.5%)	** < 0.0001**
Glycemia (mg/dL)	138 ± 59	122 ± 51	**0.013**
Creatinine (mg/dL)	1.74 ± 2.06	1.15 ± 1.29	** < 0.0001**
IMPROVEDD	2.6 ± 1.3	2.1 ± 1.2	** < 0.0001**
CHA_2_DS_2_-VASc	3.2 ± 1.4	2.6 ± 1.5	** < 0.0001**

Bold values indicates statistically significant

Table 2 reports the clinical characteristics and treatment of patients with and without thrombotic events occurred during hospitalization. Compared to the patients who did not experience thrombotic events, patients with thrombotic events were older, with an higher prevalence of former smokers, hypertension, atrial fibrillation, coronary artery disease (CAD), heart failure, peripheral vascular disease, TIA/stroke, COPD, and a higher need of intensive care unit (ICU). Furthermore, they had higher IMPROVEDD Score, creatinine, D–dimer, and lower P/F ratio and albumin. Furthermore, they have a significant higher prevalence of steroids and heparins low-molecular-weight heparins (LMWH) use (Table 2).

A bivariate logistic analysis was performed to analyze the predictors of thrombosis in COVID-19 patients; P/F ratio < 200 (OR 1.718, 95% CI 1.085–2.718, *p* = 0.021), albumin (OR 1.693, 95% CI 1.055–2.716, *p* = 0.029), D-dimer (OR 3.469, 95% CI 2.110–5.703, *p* < 0.0001), CAD (OR 1.800, 95% CI 1.086–2.984, *p* = 0.023) and heart failure (OR 2410, 95% CI 1.385–4.193, *p* = 0.002) independently predicted thrombotic events in this population (see Table 3).

**Table 3:** Multivariable logistic analysis to analyze the predictors of thrombosis in COVID-19 patients

P/F ratio < 200	OR 1.718	95% CI 1.085–2.718	**0.021**
Albumin < 35 (g/L)	OR 1.693	95% CI 1.055–2.716	**0.029**
D–dimer ≥ 1100 (ng/mL)	OR 3.469	95% CI 2.110–5.703	** < 0.0001**
CAD	OR 1.800	95% CI 1.086–2.984	**0.023**
Heart failure	OR 2.410	95% CI 1.385–4.193	**0.002**

Bold values indicates statistically significant

## Discussion

The present study shows that patients affected by COVID-19 with P/F ratio < 200 have a high risk to develop arterial and venous thrombotic events.

To the best of our knowledge, only an Italian single-center study reported a significant association between P/F ratio and thrombosis in a limited number of COVID-19 patients (*n* = 180) [18], but the interplay between P/F ratio and mechanisms of thrombosis was not investigated. The novelty of the present study is the relationship between P/F ratio and D–dimer suggesting that hypoxia may be a trigger of clotting activation. This hypothesis is in keeping with previous reports showing that hypoxemia may generate arterial and venous thrombosis [7, 19]. Thus, observational and experimental studies showed that hypoxia/hypoxemia increased the risk of thrombosis [7, 19]. Virchow’s triad (hypercoagulability, endothelial injury and stasis) seems to be implicated in this physio-pathological process [7]. Local and systemic hypoxia due to several risk factors (as cancer, high altitude, immobilization after major trauma or surgical interventions, etc.) activate the hypoxia-inducible transcription factors (HIFs) that regulate the genes that mediate coagulation and fibrinolysis [7, 19]. Thus, the HIFs activation leads to the increase of prothrombotic substances as tumor necrosis factor (TNF)-α, interleukin (IL)-1, IL-6, IL-12, plasminogen activator inhibitor (PAI)-1 [7] or to decrease anticoagulant factors as protein S [20]. All this pathological process is well summed up by some animal studies that have shown how hypoxia increases the incidence of experimental venous thrombosis in mice [7, 21].

Hypoxia is also implicated in thrombosis of the arterial district. Experimental studies in patients with peripheral arterial disease showed that when platelets are exposed to hypoxia, platelet activation proteins, P-selectin, fibrinogen binding protein expression and GpIIb/IIIa will increase [7, 22] favoring a prothrombotic state. In addition, hypoxia causes vasoconstriction favoring the formation of micro thrombosis and decreasing the share of pulmonary perfusion [23].

The association between P/F ratio and albumin is another novel finding that may provide new insight into the relationship between P/F ratio and thrombosis in COVID-19. We have previously demonstrated the prothrombotic role of serum albumin and D–dimer in patients with COVID-19 [24, 25] and that restoring serum albumin levels improves the thrombotic risk in this setting [25]. Thus, albumin is implicated in both clotting and platelet activation with several mechanisms such as inhibition of fibrin polymerization, increase of the antithrombin III action, and modulating the hepatic synthesis of factor V, factor VIII, and fibrinogen [26, 27]. Furthermore, albumin impairs platelet aggregation downregulating Nox_2_ activation, a powerful source of reactive oxidant species [28].

At this regard, it is also interesting to note that the administration of albumin before a lung transplant, administered to improve the prothrombotic state, is able to increase the P/F ratio [29]. It remains to be established, however, the association between hypoxia and albumin. Low serum albumin levels could contribute to pulmonary edema formation and to consequent hypoxia in patients with ARDS [30]. Furthermore, a previous study in hypo-proteinemic patients with ARDS showed that those treated with albumin had an increase of oxygenation as assessed by P/F ratio [31].

In accordance with previous studies, we found an association among coronary heart disease (CAD), heart failure and cardiovascular events in patients with COVID [32]. Patients affected by CAD and heart failure are more inclined to have arterial and venous thrombotic events and mortality after pneumonia [33, 34]. Increased platelet aggregation and clotting system activation, as documented by up-regulation of tissue factor and down-regulation of activated protein C, could be implicated in this process [33, 34].

The study has limitations. We reported the P/F ratio assessed at admission in the medical wards thus, this parameter could change during hospitalization. We did not stratify the population according to the COVID-19 waves therefore, different incidences of thrombosis could be present according different periods of hospitalization.

In conclusion, the study shows that P/F ratio is associated to thrombotic events in patients hospitalized for COVID-19 and suggests hypoalbuminemia as a mechanism accounting for hypoxia-related hypercoagulability.

## Data Availability

Data will be available upon reasonable request to the corresponding author.

## Abbreviations

ARDS: Acute respiratory distress syndrome
CAD: Coronary artery disease
COPD: Chronic obstructive pulmonary disease
CRP: C-reactive protein
CT: Computer tomography
EKG: Electrocardiogram
FiO2: Fraction of inspired oxygen
HIFs: Hypoxia-inducible transcription factors
ICU: Intensive care unit
IL: Interleukin
LMWH: Low molecular weight heparin
NMR: Nuclear magnetic resonance
PAI: Plasminogen activator inhibitor
PaO2: Arterial partial pressure of oxygen
PaO_2_/FiO_2_: P/F ratio
SD: Standard deviation
IQR: Interquartile range
TIA: Transient ischemic attack
TNF: Tumor necrosis factor
